# The Akt1/IL-6/STAT3 pathway regulates growth of lung tumor initiating cells

**DOI:** 10.18632/oncotarget.5626

**Published:** 2015-10-13

**Authors:** Donatella Malanga, Carmela De Marco, Ilaria Guerriero, Fabiana Colelli, Nicola Rinaldo, Marianna Scrima, Teresa Mirante, Claudia De Vitis, Pietro Zoppoli, Michele Ceccarelli, Miriam Riccardi, Maria Ravo, Alessandro Weisz, Antonella Federico, Renato Franco, Gaetano Rocco, Rita Mancini, Antonia Rizzuto, Elio Gulletta, Gennaro Ciliberto, Giuseppe Viglietto

**Affiliations:** ^1^ Dipartimento di Medicina Sperimentale e Clinica, Università Magna Graecia, Catanzaro, Italy; ^2^ Biogem scarl, Istituto di Ricerche Genetiche, Ariano Irpino (Avellino), Italy; ^3^ Dipartimento di Scienze Mediche e Chirurgiche, Università Magna Graecia, Catanzaro, Italy; ^4^ Dipartimento di Scienze e Tecnologie, Università del Sannio, Benevento, Italy; ^5^ Dipartimento di Medicina e Chirurgia, Università di Salerno, Baronissi, Italy; ^6^ Dipartimento di Dipartimento di Medicina Molecolare e Biotecnologie Mediche, Università Federico II, Napoli, Italy; ^7^ IRCCS Istituto Nazionale Tumori Fondazione G. Pascale, Napoli, Italy; ^8^ Dipartimento di Medicina Clinica e Molecolare, Università di Roma “La Sapienza” Ospedale S. Andrea, Roma, Italy; ^9^ Dipartimento di Scienze della Salute, Università Magna Graecia, Catanzaro, Italy

**Keywords:** NSCLC, tumor initiating cells, Akt1, IL-6, STAT3

## Abstract

Here we report that the PI3K/Akt1/IL-6/STAT3 signalling pathway regulates generation and stem cell-like properties of Non-Small Cell Lung Cancer (NSCLC) tumor initiating cells (TICs). Mutant Akt1, mutant PIK3CA or PTEN loss enhances formation of lung cancer spheroids (LCS), self-renewal, expression of stemness markers and tumorigenic potential of human immortalized bronchial cells (BEAS-2B) whereas Akt inhibition suppresses these activities in established (NCI-H460) and primary NSCLC cells. Matched microarray analysis of Akt1-interfered cells and LCSs identified IL-6 as a critical target of Akt signalling in NSCLC TICs. Accordingly, suppression of Akt in NSCLC cells decreases IL-6 levels, phosphorylation of IkK and IkB, NF-kB transcriptional activity, phosphorylation and transcriptional activity of STAT3 whereas active Akt1 up-regulates them. Exposure of LCSs isolated from NSCLC cells to blocking anti-IL-6 mAbs, shRNA to IL-6 receptor or to STAT3 markedly reduces the capability to generate LCSs, to self-renew and to form tumors, whereas administration of IL-6 to Akt-interfered cells restores the capability to generate LCSs. Finally, immunohistochemical studies in NSCLC patients demonstrated a positive correlative trend between activated Akt, IL-6 expression and STAT3 phosphorylation (*n* = 94; *p* < 0.05). In conclusion, our data indicate that aberrant Akt signalling contributes to maintaining stemness in lung cancer TICs through a NF-kB/IL-6/STAT3 pathway and provide novel potential therapeutic targets for eliminating these malignant cells in NSCLC.

## INTRODUCTION

Lung cancer is the leading causes of cancer-related mortality worldwide, with a 5-year survival rate of only 15% for NSCLC [1]. One of the main causes of disease relapse is the emergence of cancer cells resistant to therapy. This phenomenon has been attributed to a population of cells endowed with tumour-initiating potential that support the growth of NSCLC [2–4]. These cells, known as Tumour initiating cells (TICs), have a high capacity for self-renewal and multi-lineage differentiation and are believed to be responsible for tumor development, recurrence and dissemination as well as the acquisition of drug resistance [3]. In the experimental setting, TICs are able to grow as spheres under non-adherent, serum-free conditions and show high clonogenic growth *in vitro* and tumorigenic potential *in vivo*. TICs isolated from NSCLCs are characterised by the expression of specific markers including CD133, Oct-4 and ABCG2 [5–9]. However, the influence of specific oncogenic pathways on the growth and survival of TICs in NSCLC remains relatively unknown. Among the multiple pathways that are activated in NSCLC TICs, recent evidence indicated that the phosphatidylinositol 3-kinase (PI3K)/Akt pathway may play a role in survival and proliferation of cancer stem-like cells [10–12]. Moreover, the sustained activation of the EGFR/SRC/Akt signaling has been implicated in the regulation of self-renewal, growth and expansion of the side population compartment of NSCLC cells [13].

The serine/threonine protein kinases Akt represent the main known end-point of PI3K signaling. Akt is activated by recruitment to the cell membrane via binding of its PH domain to 3′-phosphorylated phosphatidyl-inositols generated by PI3K and subsequent phosphorylation at T308 and S473 [14, 15]. Conversely, the lipid phosphatase PTEN attenuates Akt activation by dephosphorylating the 3′ position of phosphatidyl-inositols [16].

Expectedly, among the different molecular lesions that contribute to the development of NSCLC aberrant Akt activation is a frequent event [17, 18]. Akt kinases can be activated through several mechanisms, which result from distinct and often mutually exclusive events that include activating mutations or amplification of Akt1, Akt2 and Akt3, mutations of upstream activators (KRAS, PIK3CA), or loss of upstream inhibitors (PTEN) [17, 19]. We have recently identified a somatic mutation in the gene encoding Akt1 resulting in a glutamic acid to lysine substitution at amino acid 17 (E17K) in a subset of NSCLC patients with increased Akt activity [20]. The mutant K17 residue increases the affinity for PI(4,5)P2, enhancing plasma membrane recruitment and activity [21, 22]. Accordingly, endogenous Akt1-E17K mutant detected in lung cancer cells shows enhanced membrane localization and activity [20, 21]. Nevertheless, the molecular mechanisms whereby Akt1-E17K promotes cancer in the human remain to be fully understood. Recently we have shown that Akt1-E17K mutant is able to transform lung epithelial cells [23]. In this manuscript, we provide complementary biochemical and biological evidence that aberrant signalling through the PI3K/Akt pathway, due to gain-of-function mutation in Akt1 (E17K) or PIK3CA (E545K) or to PTEN loss, regulates self-renewal of NSCLC TICs *in vitro* and tumor growth *in vivo* by activating the NF-kB/IL-6/STAT3 axis.

## RESULTS

### Activation of PI3K/Akt pathway confers increased spheroid-forming ability and highly tumorigenic potential to bronchial epithelial cells

Aberrant Akt activation is a frequent event in NSCLC that results from gain-of-function mutations of PIK3CA, loss of PTEN or activating mutations of Akt1 itself [17–19]. Here we have investigated whether and how the activated PI3K/Akt pathway influences the generation and/or stem cell-like properties of TICs. As model system we used human bronchial epithelial cells (BEAS-2B), a non-tumorigenic line that had been immortalised by infection with Adenovirus 12/SV40 hybrid virus (BEAS-2B) [35–38]. After lentiviral-transduction control BEAS-2B (BEAS-C), BEAS-Akt1-E17K, BEAS-PIK3CA-E545K and BEAS-shPTEN cells were isolated and expanded [23]. The presence of the exogenous mutant Akt1, mutant PIK3CA or endogenous PTEN proteins was detected by immunoblot ([23] and Supplemental Figure S1A, respectively). The status of the PI3K/Akt pathway was determined by analysis of AKT and/or GSK3 phosphorylation ([23] and Supplemental Figure S1A, respectively). Similarly to what described previously for mutant Akt1-E17K [23], active PIK3CA (E545K) or PTEN loss render human bronchial epithelial cells BEAS-2B tumorigenic (Figure S1B). This high tumorigenic potential suggested that activation of the PI3K/Akt pathway may affect number and properties of NSCLC TICs.

**Figure 1:**
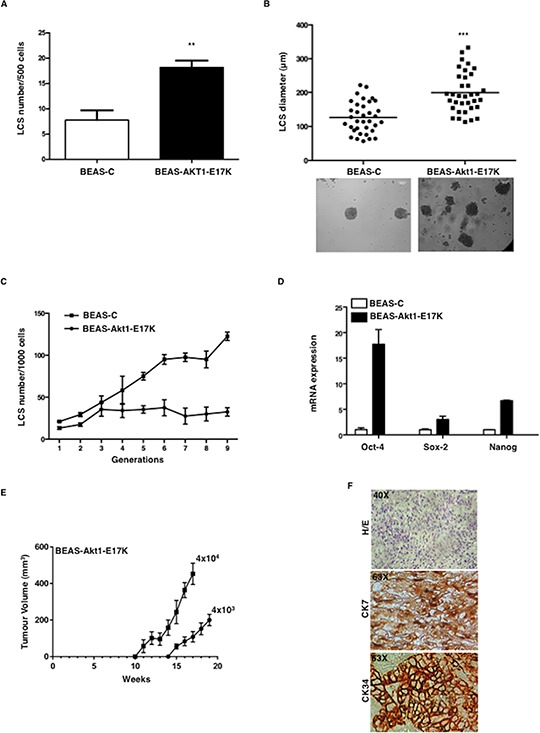
Mutant Akt1-E17K increases formation of LCSs **A.** Number of primary LCSs generated from control BEAS-2B cells or from the corresponding cells infected with pLenty-Akt1-E17K. **p < 0.01. **B.** Analysis of size distribution (μm) of LCSs generated from control BEAS-C and BEAS-Akt1-E17K cells by phase-contrast microscopy ***p < 0.001. **C.** Number of LCSs generated from control BEAS-C and BEAS-Akt1-E17K cells during serial passages expressed as mean ± SD. **D.** Relative mRNA expression of stemness genes by Q-RT-PCR in BEAS-C and BEAS-Akt1-E17K cells. **E.** Tumor growth of primary LCS generated from BEAS-Akt1-E17K cells (4 × 10^3^, 4 × 10^4^), injected into the flank of NOD/SCID mice (*n* = 8/group); data are shown as mean ± SD. **F.** Representative images of CK7 and CK34 immunostaining of tumours generated from single cell suspensions of primary LCSs derived from BEAS-Akt1-E17K cells subcutaneously injected into the flank of NOD/SCID and explanted 15–20 weeks after injection. Magnification as indicated.

To investigate the role of aberrant PI3K/Akt signalling in NSCLC TICs, BEAS-C, BEAS-Akt1-E17K, BEAS-PIK3CA-E545K and BEAS-shPTEN cells were cultured in low adhesion conditions in sphere medium. Mutant Akt1 in BEAS-2B cells produced a pronounced increase in the number of LCSs (Figure 1A), with virtually all LCSs larger than 100 μm (Figure 1B). In addition, while BEAS-C gave rise to constant LCS number throughout the generations in serial propagation assays (~20 out of 10^3^ plated cells, 2% on average), the expression of mutant Akt1-E17K induced a marked increase in the number of LCS-forming cells over passages from 20/10^3^ plated cells at passage 1 to 120/10^3^ plated cells at passage 9 (Figure 1C), which was paralleled by an increase in the expression of mRNA encoding stemness-related markers such as Oct-4, Nanog and Sox2 (Figure 1D). Finally, we found that BEAS-Akt1-E17K LCSs were able to efficiently sustain tumor growth *in vivo*. Serial dilutions of BEAS-C and BEAS-Akt1-E17K cells grown as LCSs (4 × 10^3^, 4 × 10^4^) were injected subcutaneously into NOD/SCID mice and monitored for tumor appearance up to ~7 months. Mice injected with BEAS-C LCSs (4 × 10^3^, 4 × 10^4^) yielded no tumor (*n* = 8 mice/group) whereas LCSs derived from BEAS-Akt1-E17K cells (4 × 10^3^, 4 × 10^4^) promoted formation of poorly differentiated carcinomas positive for cytokeratins (CK7, CK34) in 7/8 and 8/8 mice, respectively (Figure 1E, 1F). No tumor was detected in mice injected with the same numbers (4 × 10^3^, 4 × 10^4^) of BEAS-C or BEAS-Akt1-E17K grown in adherent conditions.

Results for mutant PIK3CA or PTEN loss are reported in Figure S2. Similarly to BEAS-Akt1-E17K cells, BEAS cells expressing mutant PIK3CA or silenced for PTEN showed a pronounced increase in the number and size of LCSs generated, expressed consistently higher mRNA levels of Oct-4, Nanog and Sox2, and were able to efficiently sustain tumor growth *in vivo* as LCSs at low number (Supplemental Figure S2A–S2E).

Altogether, these results indicate that aberrant signalling through the PI3K pathway – induced by mutant Akt1, PIK3CA or by PTEN loss - significantly increases the percentage of cells able to initiate *in vitro* growth as spheroids enriched in TICs that efficiently support tumor growth *in vivo*.

### Inhibition of Akt signalling impairs formation and maintenance of LCSs

As a complementary approach to assess the importance of Akt1 signalling in NSCLC TICs we suppressed Akt1 expression in an established lung cancer cell line (NCI-H460) that harbours an activating mutation of PIK3CA (E545K) and in one primary short-term culture (PEd/10) derived from pleural effusions of lung cancer patients that engraft very efficiently (10–16 weeks latency) giving rise to adenocarcinoma [24].

**Figure 2:**
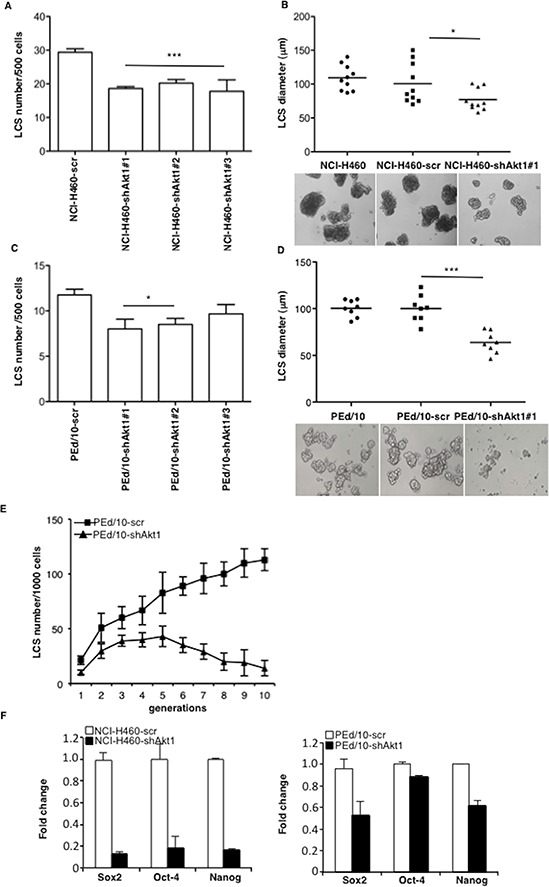
Akt1 regulates formation and maintenance of NSCLC LCS **A.** Number of primary LCSs generated from NCI-H460 cells or the corresponding cells infected with lentivirus expressing shRNAs to Akt1 (clones #1, #2, #3, respectively). Data are shown as mean ± SD. ***p < 0.001. **B.** Analysis of size distribution (μm) of LCSs generated from NCI-H460 or NCI-H460-shAkt1 cells by phase-contrast microscopy *p < 0.05. **C.** Number of primary LCSs generated from PEd/10 cells or the corresponding cells interfered for Akt1 (clones #1, #2, #3, respectively) *p < 0.05. **D.** Analysis of size distribution (μm) of LCSs generated from PEd/10 cells by phase-contrast microscopy ***p < 0.001. **E.** Representative analysis of primary LCSs generated from PEd/10 cells or the corresponding cells interfered for Akt1 during serial passages (*n* = 10). **F.** Left, relative mRNA expression of stemness genes by Q-RT-PCR in NCI-H460 cells and derivatives; right, relative mRNA expression of stemness genes by Q-RT-PCR in PEd/10-scr cells and derivatives.

NCI-H460 and PEd/10 cells were transduced with lentivirus expressing three different shRNAs (clones #1, #2 and #3) to Akt1. Silencing of Akt1 in NCI-H460 and PEd/10, as assessed by immunoblot (Supplemental Figure S3A), resulted in reduced expansion of tumors generated by NSCLC cells injected into immunodeficient mice (*n* = 5/group) (Supplemental Figure S3B and S3C, respectively), indicating that Akt1 plays a significant role in the malignant behaviour of NSCLC cells. Suppression of Akt1 in NCI-H460 cells produced a pronounced decrease in the number and size of LCSs compared to control cells (Figure 2A). Most LCSs (~50%) generated by parental NCI-H460 or NCI-H460-scr, were larger than 100 μm, differently from those generated by NCI-H460-shAkt1 (Figure 2B). Similar results were obtained in primary NSCLC cells PEd/10 (Figure 2C and 2D). In addition, whereas the number of PEd/10-scr LCS-forming cells increased steadily throughout the generations in self-renewal assays (from approximately 2% to more than 10%), the suppression of Akt1 expression prevented the increase in the number of LCS-forming cells over 10 passages (Figure 2E). Accordingly, Akt1 knock-down also reduced mRNA expression of stem cell markers in NCI-H460 and PEd/10 cells (Figure 2F left and right, respectively).

**Figure 3:**
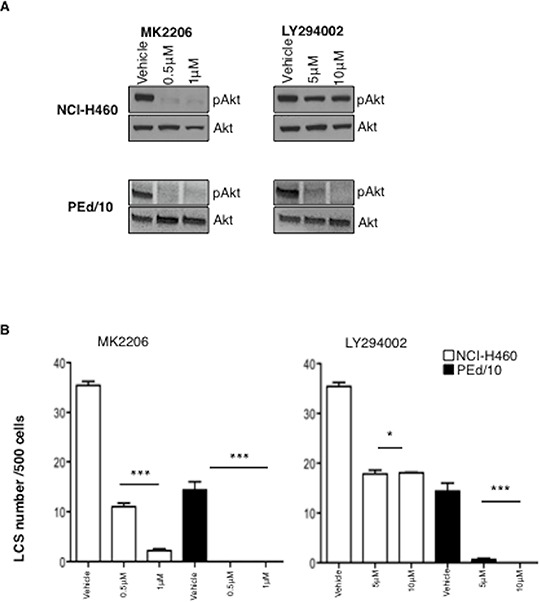
Akt signalling is required for maintenance of NSCLC LCSs **A.** Immunoblot analysis of Akt phosphorylation status in NCI-H460 and PEd/10 cells treated with MK-2206 and LY294002 at the indicated concentrations. **B.** Analysis of primary LCSs generated from NCI-H460 and PEd/10 cells treated with MK 2206 and LY294002 at the indicated concentrations. ***p < 0.001, **p < 0.01, *p < 0.05.

Since the knock-down of Akt1 did not completely abrogate the capability to form LCSs, we reasoned that in NSCLC cells Akt1 function could be partially complemented by other Akt isoforms. Of these, Akt2 expression is ubiquitous whereas Akt3 shows a more restricted expression pattern [39]. Therefore, we decided to interfer NCI-H460 and PEd/10 cells with lentivirus expressing shRNA to Akt2, to determine the relative effects of Akt1 and/or Akt2 signalling in NSCLC TICs. Similarly to Akt1 suppression, also the silencing of Akt2 in NCI-H460 and PEd/10 cells produced a decrease in the number and size of LCSs (Supplemental Figure S4A–S4F). However, we were not able to obtain complete suppression of both isoforms, thus the double interfered cells could not be used to determine the effects of complete suppression of Akt signalling in NSCLC TICs (not shown). Therefore, we used a pharmacological inhibitor of all Akt isoforms (MK2206) and an inhibitor of the most important upstream activator of Akt (LY294002) to determine the effects of Akt signalling in NSCLC TICs. We found that exposure of NSCLC cells to MK2206 or LY294002 suppressed and/or decreased Akt phosphorylation (Figure 3A) and significantly impaired in a dose-dependent manner spheroid-forming capability of NSCLC cells (Figure 3B, left and right respectively). It is of note that the established cell line NCI-H460 was less sensitive than the primary PEd/10 cells to both inhibitors, and in particular to LY294002, possibly because these cells carry a constitutively activated allele of PIK3CA.

These results suggested that the modulation of Akt activity is able to regulate the percentage of NSCLC cells able to initiate growth as spheroids, which are apparently enriched in putative TICs.

### IL-6 is a critical target of Akt signalling in NSCLC TICs

We proceeded to identify the downstream targets of constitutive Akt1 signalling that regulate generation and/or maintenance of LCSs. To this aim, we first identified genes that are regulated by Akt1 by comparing the transcriptome of NCI-H460 cells with that of NCI-H460-shAkt1. We identified 336 differentially regulated transcripts (DEGs) (fold change cut-off: 1.5; *p* < 0.05), of which 161 were up-regulated and 175 were down-regulated (“Akt1 signature”, listed in Supplemental Table S1). Then we identified genes that are implicated in the generation and/or maintenance of LCSs by comparing the transcriptome of NCI-H460 cells grown as LCSs with that of NCI-H460 cells grown in adherent condition. As listed in Supplemental Table S2 (“LCS signature”) we identified 1715 DEGs (fold change cut-off: 1.5; *p* < 0.05), of which 909 were up-regulated and 806 were down-regulated. To determine what subset of Akt-regulated genes was implicated in the generation and/or maintenance of NSCLC LCSs, we matched the “Akt1 signature” with the “LCS signature”. As shown in the Venn diagram of Supplemental Figure S5A, we identified 152 DEGs that are common between Akt1-interfered and LCS-derived NCI-H460 cells (see Supplemental Table S3). Analysis of DEGs indicated that several genes (i.e. cytokines IL-2, IL-6, IL-8, IL-1) known to be involved in migration, metastasis and stem cell maintenance, are apparently co-regulated in Akt1-interfered NCI-H460 and in LCSs (see Supplemental Tables S1–S3 and Heat Map in Supplemental Figure S5B). For the rest of the manuscript we have focused our attention on IL-6 because this gene presented the most consistent modulation in the matching of “Akt1 signature” and “LCS signature” as shown in Supplemental Figure S5B. The array data are loaded into ArrayExpress with accession number E-MTAB-1699.

**Figure 4:**
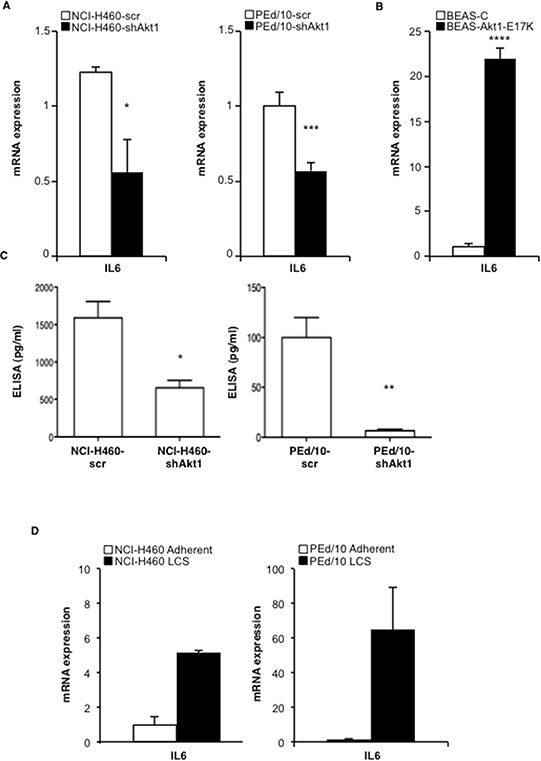
Akt1 regulates IL-6 expression in NSCLC cells **A.** Relative mRNA expression of IL-6 by Q-RT-PCR in NCI-H460 cells, PEd/10, and derivatives. *p < 0.05, ***p < 0.001. **B.** Relative mRNA expression of IL-6 by Q-RT-PCR in BEAS-2B and derivatives ****p < 0.0001. **C.** Levels of secreted IL-6 by ELISA in NCI-H460 cells and derivatives (1,500 and 635 pg/ml, respectively), in PEd/10 cells and derivatives (100 pg/ml and 5 pg/ml, respectively) *p < 0.05, **p < 0.01. **D.** Q-RT-PCR analysis of mRNA expression of IL-6 in adherent and LCSs generated from NCI-H460 and PEd/10.

We confirmed that IL-6 expression is down-regulated in NSCLC cells interfered for Akt1 by quantitative RT-PCR (Figure 4A, left and right panels) but is up-regulated in BEAS-2B cells expressing Akt1-E17K, PIK3CA-E545K or shPTEN (Figure 4B and Supplemental Figure S5C, respectively). NCI-H460 cells and PEd/10 cells interferred for Akt1 showed a marked reduction of IL-6 secretion (Figure 4C). Notably, IL-6 is expressed at higher levels in LCSs than in adherent cells (Figure 4D). The finding that IL-6 receptor (IL-6r) and gp130 mRNAs were detected in all cellular systems under study (28 and 26 number of cycles respectively, Supplemental Figure S5D) indicated that human lung cells may be responsive to secreted IL-6. Taken together these results indicate that Akt1 regulates the IL-6/IL-6r signalling axis, and that this pathway is significantly up-regulated in LCSs.

**Figure 5:**
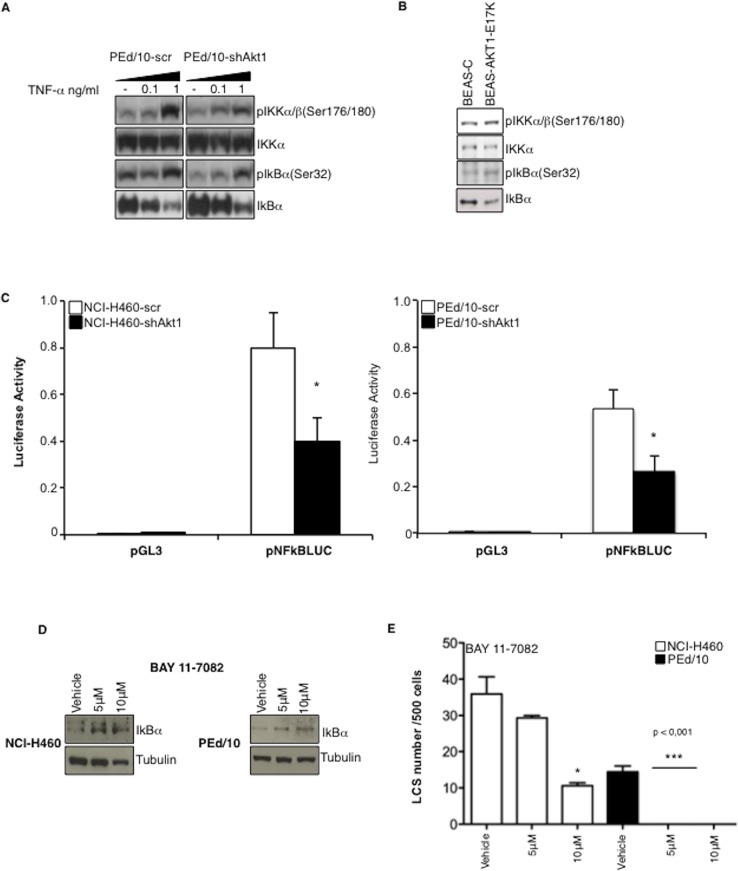
Akt1 regulates NF-kB activity in NSCLC cells **A.** Immunoblot analysis of members of the NF-kB pathway in PEd/10 and derivative cells treated with increasing doses of TNF-α (0.1, 1 ng/ml). **B.** Immunoblot analysis of members of the NF-kB pathway in BEAS-C and BEAS-Akt1-E17K cells. **C.** Luciferase assay of NF-kB activity in NCI-H460, PEd/10 cells and derivatives interfered for Akt1 transfected with a reporter containing a NF-kB-responsive promoter, *p < 0.05. **D.** Immunoblot analysis of IkBα in NCI-H460 and PEd/10 cells treated with BAY11-7082. **E.** Analysis of spheroid forming capability of NCI-H460 and PEd/10 cells treated with BAY11-7082, *p < 0.05, ***p < 0.001.

IL-6 expression is stimulated by NF-kB, whose activity is regulated by Akt through phosphorylation of IkB kinases (IkKs). Accordingly, we found that interference with Akt1 decreased phosphorylation of IkKα/β (S176 /180) and of IkB (S32), thus resulting in a 2-fold stabilization of IkB (Figure 5A, lane -). The effect of suppressing Akt1 in PEd/10 cells was more evident after treatment of PEd/10-scr and PEd/10-shAkt1 cells with increasing doses of TNF-α (0.1, 1 ng/ml) (Figure 5A). Conversely, expression of Akt1-E17K in BEAS-2B cells increased phosphorylation of both IkKα/β and IkB, resulting in the destabilization of IkB (Figure 5B). The resulting stabilization of IkB in NCI-H460-shAkt1 and PEd/10-shAkt1 was associated with decreased NF-kB transcriptional activity as evaluated by the measure of NF-kB activity in reporter assays (Figure 5C, left and right panels, respectively). Finally, in agreement with the hypothesis that NF-kB activity is required for spheroid generation, we found that an IkK inhibitor (BAY11-7082), which induces IkB stabilization, strongly reduced formation of LCSs (Figure 5D–5E). It is worth noting that, as in the case of Akt or PI3K inhibitors, NCI-H460 cells were less sensitive to BAY11-7082 than the primary PEd/10 cells.

### IL-6/IL-6 receptor axis is necessary for NSCLC cells to form LCSs and tumors

**Figure 6:**
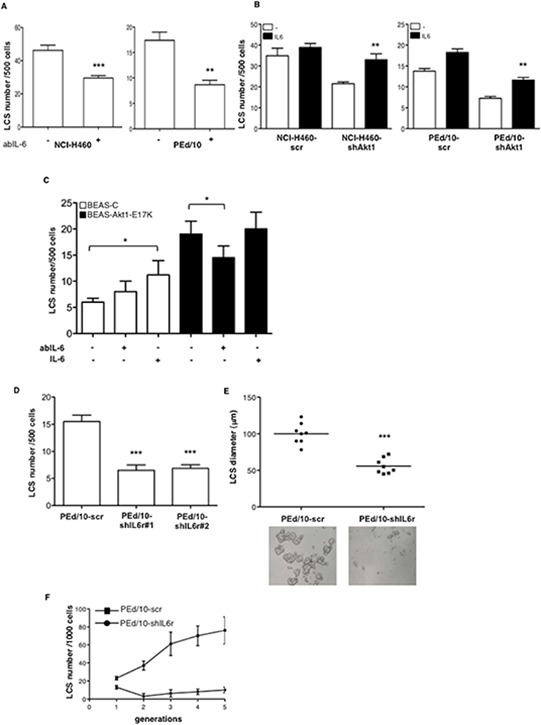
IL-6 sustains formation of NSCLC LCSs **A.** Effects of anti-IL-6 mAb (3.0 μg/ml) on the formation of secondary LCSs in NCI-H460 and PEd/10 cells. ***p < 0.001; **p < 0.01. **B.** Effects of IL-6 administration (20 ng/ml) on the formation of secondary LCSs in NCI-H460 and derivatives, PEd/10 and derivatives. **p < 0.01. **C.** Effects of anti-IL-6-mAb (3.0 μg/ml) or IL-6 administration (20 ng/ml) on the formation of LCSs generated from BEAS-C and BEAS-Akt1-E17K. *p < 0.05. **D.** Number of LCSs generated from PEd/10 cells or the corresponding cells interfered for IL-6r (PEd/10-shIL-6r; clones #1, #2). ***p < 0.001. **E.** Analysis by phase-contrast microscopy of size distribution (μm) of LCSs generated from PEd/10-scr and PEd/10-shIL-6r cells, ***p < 0.001. **F.** Number of LCSs formed from PEd/10-scr and PEd/10-shIL-6r cells during serial passages (*n* = 5).

Subsequently, we investigated the functional role of IL-6 in the generation and maintenance of NSCLC LCSs, by exposing secondary NCI-H460 and PEd/10 LCSs to a blocking mAb to IL-6 (anti-IL-6). We found that anti-IL-6 markedly reduced the capability of NCI-H460 and PEd/10 cells to generate secondary spheres (Figure 6A, left and right panels, respectively). Conversely, we observed that administration of IL-6 to Akt1-interfered NCI-H460 and PEd/10 cells, restored the capability to generate LCSs (Figure 6B, left and right panels, respectively). Similarly, we found that treatment of BEAS-Akt1-E17K cells with anti-IL-6 reduced their spherogenic capability whereas treatment of BEAS-C cells with IL-6 increased the number of secondary LCSs (Figure 6C).

In agreement with these results the knock-down of IL-6r through use of two different shRNAs (clones #1 and #2) (Supplemental Figure S6A) induced a pronounced decrease in the number (Figure 6D) and size (Figure 6E) of LCSs generated by PEd/10 cells. In addition, whereas in PEd/10-scr cells the number of LCS-forming cells increased steadily throughout the generations in self-renewal assays, the suppression of IL-6r strongly prevented the increase in the number of LCS-forming cells, abolishing it completely after 5 passages (Figure 6F).

Altogether, these results indicated that the IL-6/IL-6r axis plays a significant role downstream Akt1 signalling in human bronchial epithelial cells and that it contributes to the malignant behaviour of NSCLC cells.

### STAT3 regulates efficiency of LCS formation and tumorigenicity of NSCLC cells

**Figure 7:**
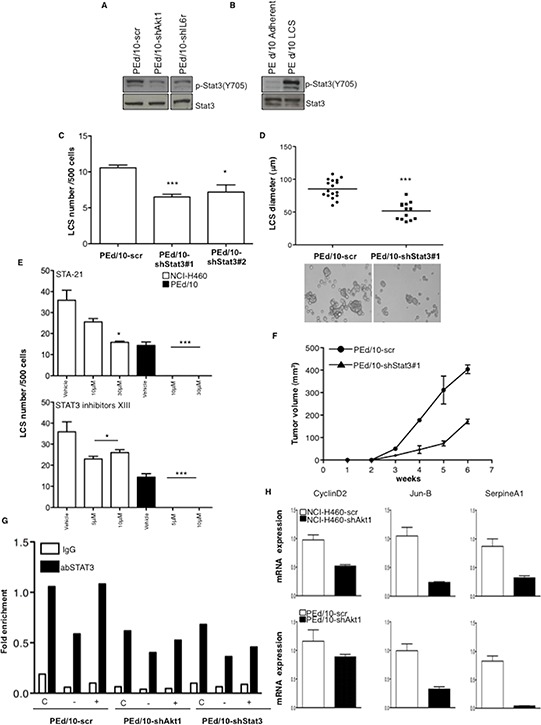
LCS formation and tumorigenicity of NSCLC cells are regulated through STAT3 activity **A.** Immunoblot analysis of phosphorilated and total STAT3 (pY705) in PEd/10-scr, PEd/10-shAkt1 and PEd/10-shIL-6r cells. **B.** Immunoblot analysis of phosphorylated (pY705) and total STAT3 in PEd/10-scr cells grown as LCS or in adherent conditions. **C.** Number of primary LCSs generated from PEd/10 cells or the corresponding cells interfered for STAT3 (PEd/10-shSTAT3; clones #1, #2). ***p < 0.001, *p < 0.05. **D.** Analysis of size distribution (μm) of LCSs generated from PEd/10 cells and derivatives by phase-contrast microscopy ***p < 0.001. **E.** Analysis of spheroid forming capability of NCI-H460 cells and PEd/10 cells in the presence of Sta-21 and Stat3 Inhibitor XIII at the indicated concentrations (upper and lower panels, respectively). ***p < 0.001, *p < 0.05. **F.** Tumor growth of PEd/10-scr and the corresponding cells interfered for STAT3 injected into immunodeficient mice (*n* = 5/group). **G.** STAT3 occupancy (fold enrichment) of JunB promoter as determined by Chip of cross-linked DNA extracted from control PEd/10-scr, PEd/10-shAkt1 and PEd/10-shSTAT3 cells growing in complete (C), serum free (−) and serum free plus IL-6 (+) medium using anti-STAT3 or control IgG isotype antibodies. **H.** Relative mRNA expression of STAT3 target genes in PEd/10-scr and PEd/10-shAkt1 cells by Q-RT-PCR analysis.

The latent transcription factor Signal Transducer and Activator of Transcription 3 (STAT3) is a pivotal downstream mediator of IL-6 signalling that plays a role in stem cells and tumorigenicity [40, 41]. Thus, we examined the status of STAT3 activation in NSCLC cells interfered for Akt1 or IL-6r. Suppression of Akt1 and/or IL-6r in NSCLC cells strongly decreased the levels of phosphorylated STAT3 (Y705) (Figure 7A). On the other hand, in keeping with a role for STAT3 in TICs, phosphorylated STAT3 was greater in LCSs than in the corresponding adherent cells (Figure 7B). Silencing of STAT3 with two different shRNAs (Supplemental Figure S6B, clones #1 and #2, respectively) or pharmacological inhibitors (Sta-21 and Stat3 Inhibitor XIII) produced a significant decrease in the number and in the size of LCSs generated by NSCLC cells (Figure 7C–7E) and impaired tumor growth in mice (*n* = 5/group) (Figure 7F).

Finally, we performed chromatin immunoprecipitation (ChIP) assay to investigate STAT3 activity in PEd/10-scr and the corresponding cells interfered for Akt1. Chromatin was immunoprecipitated with anti-STAT3 or rabbit IgG antibodies from PEd/10-scr and PEd/10-shAkt1 cells grown in starvation medium or complete medium in the presence or absence of IL-6 and DNA from the JunB promoter was amplified by PCR. We found that STAT3 promoter occupancy was reduced of 40% in PEd/10-shAkt1 and of 32% in PEd/10-shSTAT3 cells compared with PEd/10-scr cells in complete medium (Figure 7G). Accordingly, mRNA expression of three known STAT3 targets (Cyclin D2, JunB and SerpineA1) [42, 43] was significantly reduced in NSCLC cells interfered for Akt1 (Figure 7H).

Altogether, these results indicate that STAT3 plays a significant role downstream Akt1 signalling in human malignant NSCLC cells.

### Akt activation correlates with IL-6 and STAT3 activity in primary NSCLC

**Figure 8:**
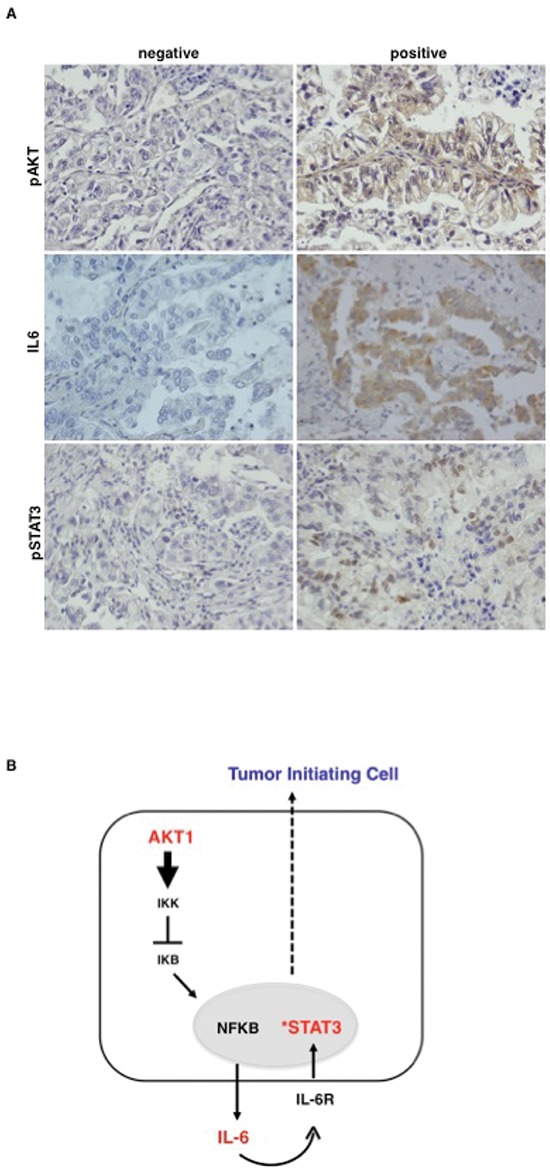
Akt activation correlates with activated STAT3 and IL-6 level in primary NSCLC **A.** Representative images of immunohistochemical analysis of TMAs of human primary lung cancer for pAkt (S473), IL-6 and pSTAT3 (Y705). Magnification 40X. **B.** The cartoon shows the Akt1/NF-kB/IL-6/STAT3 signalling pathway operating in NSCLC TICs.

Finally, we went back to human tissues and analysed the activation status of the Akt1/IL-6/STAT3 pathway in human NSCLC (*n* = 104), arrayed onto TMAs (TMA-LC1, TMA-LC2) as described [19]. Read-out were: the phosphorylation status of S473 for activated Akt (pAkt), the phosphorylation status of Y705 for activated STAT3 (pSTAT3), immunostaining with anti-IL-6 antibodies for IL-6 levels. Of 104 cases arrayed on the TMAs (49 ADC, 34 SCC, 21 different histotypes), 96 could be properly analysed for pAkt, 102 for IL-6 and 99 for pSTAT3. Simultaneous staining for all proteins was available in 94 samples. As control, 23 matched normal samples were used. Evaluation criteria are reported in Materials and Methods. Results are summarized in Supplemental Table S4. Representative stainings are shown in Figure 8A. As expected from previous work from our lab and literature, we did not find activation of Akt and STAT3 and IL-6 expression in most of the 23 control tissues (not shown). Akt activation was observed in 32 out of 96 NSCLC (13/45 ADC, 11/32 SCC, 8/19 tumors with other histotypes, respectively) as already reported [19]; IL-6 expression was observed in 38 out of 102 NSCLC analysed (18/49 ADC, in 13/34 SCC and in 7/19 tumors with other histotypes); STAT3 activation was observed in 36 out of 99 NSCLC analysed (19/46 ADC, 10/33 SCC, 7/20 tumors with other histotypes).

Tables 1 and 2 summarize the results of single and multiple correlations among Akt activation, IL-6 expression and STAT3 activation. We found a significant trend that correlated activated Akt (pAkt) and the combined status of IL-6 positive/pSTAT3 positive (*n* = 94; *p* = 0.044) and between the combined status of pAkt/IL-6 positive and pSTAT3 (*n* = 94; *p* = 0.029). In fact, 34/44 (79%) NSCLC patients that were IL-6 negative/pSTAT3 negative were also negative for pAkt whereas 7/16 (41%) NSCLC patients that were IL-6 positive/pSTAT3 positive were also positive for pAkt. These data suggest that activation of the PI3K/Akt pathway is apparently required for the synthesis of IL-6 and the activation of STAT3, though not sufficient in all cases.

**Table 1:** Correlation among phosphorylated Akt1, phosphorylated STAT3 and expression of IL6 in NSCLC patients

	pAkt negative	pAkt positive^a^
**IL-6/pSTAT3 negative**	34	9
**IL-6/pSTAT3 positive**^b,c^	10	7
**Total**	44	16

a Akt activation was evaluated with phospho-specific antibodies (pS473) and scored as negative (<10% of the tumour cells with weak, focal immunopositivity or absence of staining) and positive (>10% of tumour cells with strong or diffuse immunopositivity).

b IL-6 was evaluated with specific antibodies and scored as negative (<10% of the tumour cells with weak or absence of staining) and positive (>10% of tumour cells with strong or diffuse immunopositivity).

c STAT3 activation was evaluated with phospho-specific antibodies (Y705) and scored as negative (<5% of the tumour cells with weak or absence of staining) and positive (>5% of tumour cells with strong or diffuse immunopositivity).

*n* = 94; *p* = 0.044

**Table 2:** Correlation of phosphorylated Akt1 with IL-6 expression and phosphorylated STAT3 in NSCLC patients

	pSTAT3 negative	pSTAT3 positive^c^	Total
**Akt negative/IL-6 negative**	34	9	43
**Akt positive/IL-6 positive**^a, b^	9	6	15
**Total**	43	15	58

a See Table 1.

b See Table 1.

c See Table 1. *n* = 94; *p* = 0.029

On the other hand, of the 43 tumors that were pAkt negative/IL-6 negative, 79% (34/43) were also negative for pSTAT3 whereas 40% (6/15) of pAkt positive/IL-6 positive tumors were also positive for pSTAT3, indicating that the loop Akt/IL-6 may represent one of the mechanisms that lead to activation of STAT3 in NSCLC.

## DISCUSSION

NSCLC originates from a population of cells endowed with tumor-initiating potential denoted TICs [2, 3]. TICs isolated from NSCLCs are defined functionally for their ability to grow as spheres *in vitro* and for their tumorigenic potential *in vivo* [5, 6, 8, 9]. In this manuscript we report that aberrant activation of PI3K/Akt pathway plays a critical role in the proliferation, self-renewal and tumorigenicity of NSCLC TICs by establishing an autocrine IL-6/IL-6r signalling loop that activates STAT3 (Figure 8B). The data obtained in the *in vitro* experiments with cultured NSCLC cells were confirmed by the correlative studies in NSCLC tissues.

The first relevant observation of this manuscript is that aberrant activation of Akt - achieved through gain-of-function mutations of Akt1, PIK3CA or by loss of PTEN - results in an increase of number and self-renewal capability of TICs in immortalised human bronchial epithelial cells and consequent acquisition of a high tumorigenic potential. These findings indicate that the molecular alterations that activate Akt signalling are able to increase TIC compartment in NSCLC and are consistent with previous studies that have revealed preferential activation of the PI3K/Akt pathway in cells inside spheres and a role for PI3K signalling in survival and proliferation of cancer stem-like cells [11, 12]. Accordingly, nuclear localized Akt as been shown to enhance breast cancer stem-like cells through inactivation of cell cycle inhibitors p21 and p27 or through FoxO-Bim pathway [44, 45].

The results of self-renewal experiments indicate that active Akt signalling may shift the balance between asymmetric to symmetric division, as recently suggested to occur in breast cancer cells [46]. On the other hand, the critical role of Akt1 in TICs was confirmed by shRNA experiments in established and primary NSCLC cells, in which Akt1 inhibition suppressed LCS formation, self-renewal capacity, expression of stemness-related markers and *in vivo* tumorigenic potential. However, it is to be noted that the experiments with Akt2 shRNAs or pharmacological inhibitors, indicate that aberrant signalling through the PI3K/Akt pathway in NSCLC TICs is funnelled through the diverse Akt isoforms, and thus that efficient suppression of all isoforms is required for the eventual eradication of NSCLC TICs.

A second notable finding of our work is the identification of critical targets of constitutive Akt1 signalling that contribute to maintain cancer stem-like properties of NSCLC TICs. Accordingly, IL-6 turned out to be a critical effector of activated Akt1 in NSCLC TICs. In fact, although the most prominent role of IL-6 is to regulate immune and inflammatory responses [47], IL-6 has also been implicated in the regulation of growth, drug resistance and metastatic spread of tumors [48, 49]. The findings reported here extend previous observations that IL-6 is overexpressed in lung cancer [50, 51] and point to activation of PI3K/Akt pathway as one of the causes of the observed IL-6 overproduction. In fact we report that Akt1 suppression by shRNA in NSCLC cells decreases IL-6 levels whereas active Akt1, active PIK3CA and/or loss of PTEN increase IL-6 levels.

Moreover, the finding that IL-6 was, among the genes down-regulated in NCI-H460 cells interfered for Akt1, the DEG that resulted most consistently up-regulated in NSCLC LCSs, suggested that it plays a role in mediating the effects exerted by Akt1 on NSCLC TICs. Indeed experiments in this manuscript indicated that the production of IL-6 induced by activated Akt1 is able to promote self-renewal and tumorigenicity of NSCLC TICs. In fact, exposure of LCSs from BEAS-Akt1-E17K or from NSCLC cells to blocking anti-IL-6 mAbs markedly reduced the capability of these cells to generate LCSs; conversely, administration of IL-6 to BEAS-2B cells or to NSCLC cells interfered for Akt1, restored the capability to generate LCSs. These results are in agreement with previous studies showing that IL-6 promotes the recruitment of tumor cells into the metastatic niche [52], regulates self-renewal of breast cancer TICs in mammospheres and mediates the conversion of differentiated cancer cells in cancer stem-like cells [53–55].

The observation that LCS formation, self-renewal capacity and tumorigenic potential of NSCLC cells were markedly impaired by inhibition of IL-6 receptor, pointed to an epithelial cell-specific action of IL-6. This finding is of particular interest in the light of the fact that genes that are frequently mutated in NSCLC (i.e. EGFR, KRAS) rely on PI3K/Akt to convey their oncogenic effects [56, 57], and suggest that IL-6/IL-6r may be a general downstream effector of several oncogenes in NSCLC TICs. Accordingly, recent work indicated that mutant EGFR induces resistance to irreversible EGFR inhibitors in NSCLC through the activation of IL-6 receptor/JAK1/STAT3 signaling [58]. The results described here in human, complement also data from murine models of lung cancer, which have shown that IL-6/STAT3 signaling facilitates cancer progression by promoting cell growth [59, 60].

As to the mechanism, our data indicate that Akt regulates IL-6 production in NSCLC cells by inducing IkB degradation, which allows NF-kB to enter the nucleus and activate IL-6 transcription [61, 62] and, on the other hand, that a critical effector of IL-6 is STAT3. In fact, interference with Akt1- or IL-6-dependent signalling in NSCLC cells impairs STAT3 phosphorylation (Y705) and activity whereas active Akt1 up-regulated them. Second, interference with Akt1-dependent signalling impairs occupancy of STAT3 targets' promoters and modulates their mRNA expression. Third, STAT3 is strongly hyperactivated in NSCLC LCSs compared with cells grown in adherence. Fourth, interference with STAT3 impairs baseline and IL-6-dependent LCS formation, self-renewal capacity and tumorigenic potential. Finally, STAT3 is constitutively activated in 30–40% of NSCLC, with a positive correlation with pAkt/IL-6 positivity. These findings extend previous work demonstrating STAT3 activation in a number of cancers of epithelial origin [63] including NSCLC [64] and may provide a molecular mechanism to explain how constitutively activated STAT3 contributes to the development of lung cancer [65]. Accordingly, an IL-6/STAT3 pathway was preferentially active in CD44+/CD24- breast cancer stem cells [66] and PTEN was shown to negatively regulate TIC manteinance through STAT3-dependent signalling [67]. It is of note that STAT3 inhibition abrogate IL-6-dependent conversion of ALDH (low) prostate cancer cells to ALDH(high) [55] whereas STAT3 activation induces a melanoma-initiating cell phenotype that could favor chemotherapy resistance and relapse [68].

Moreover, one of the STAT3 targets analysed in this work, Cyclin D2, has recently been reported to be expressed in glioblastoma TICs and to regulate their growth *in vivo* [69].

Finally the TMA data suggest that activation of the PI3K/Akt/IL-6 axis may represent one mechanism that lead to activation of STAT3 in NSCLC, though not sufficient to account for all cases. Accordingly, recent proteomic results have demostrated that PI3K-dependent STAT3 activation may be also mediated by other molecules such as the tyrosine kinase expressed in hepatocellular carcinoma kinases (TEC) [70]. Such alternative mechanisms of STAT3 activation may account for the STAT3-positive tumors that are negative for Akt.

In conclusion the results presented here suggest that, among the multiple pathways that are activated in NSCLC, aberrant PI3K/Akt signalling contributes to promote and/or maintain NSCLC TICs by eliciting an autocrine IL-6/IL-6r loop that activates the transcription factor STAT3. These data provide novel potential therapeutic targets, in agreement with recent work showing that novel theanine derivatives may have therapeutic applications in the treatment of lung cancers by targeting EGFR/VEGFR-Akt/NF-κB pathways [71].

## MATERIALS AND METHODS

### Cell culture

BEAS-2B cells were purchased from LGC Standards (Milan, IT) and cultured in bronchial epithelial cell growth medium (BEBM) supplemented with growth factors (BEGM) (Cambrex Bio-science, Walkersville, MD). NCI-H460 cells were purchased from ATCC-LGC Promochem (London, UK). Primary lung cancer cells PEd/10 were derived from malignant pleural effusions of adenocarcinoma patients [24]. NCI-H460, PEd/10 cells were cultured in RPMI-1640 (Invitrogen, Carlsbad, CA), supplemented with 10% of fetal bovine serum and 100 units/ml penicillin-streptomycin (Life Technologies, London, UK).

### shRNA and viral infection

The shRNA for human Akt1 (NM_005163), Akt2 (NM_001626) IL-6r (NM_000565), STAT3 (NM_003150) and the Mission non-target control transduction virus (Sigma SHC002V) were from the Mission Program shRNA (Sigma-Aldrich, St. Louis, MO). pLenti-DEST-6.2-Akt1-E17K vector was obtained by Gateway Technology (Invitrogen). Lentiviral particles were packaged in HEK293T cells. Cells were transduced by spin infection selected with 1 μg/ml puromycin and 5 μg/ml blasticydin (Invitrogen).

### Enrichment of LCSs

LCSs were obtained by plating 10,000 cells/ml in serum-free DMEM-F12 medium (Gibco-Invitrogen) containing 10 μg/ml insulin (Sigma-Aldrich, St. Luis, MO), 1% Albumin Bovine Fraction V (Sigma-Aldrich) 50 ng/ml EGF and 25 ng/ml bFGF (PeproTech, London, UK) (sphere medium) in ultra-low attachment flasks. LCSs were expanded by trypsinization and mechanical dissociation followed by re-plating of single cell suspensions (10,000 cells /ml) in fresh sphere medium. Self-renewal of LCSs was tested by assessing the capacity of primary LCSs to generate secondary LCSs after trypsin disaggregation. Secondary LCSs were assessed at day 7 for NCI-H460 and BEAS-2B cells and at day 12 for PEd/10 cells.

Anti-IL-6 mAb and recombinant human IL-6 were purchased from PeproTech. Cells were treated with LY294002 (Sigma-Aldrich), MK2206 (Selleckchem, Huston, TX), BAY11-7082 (Sigma-Aldrich), STA-21 (Enzo Life Science, Firenze, Italy) and STAT3 Inhibitor XIII (Calbiochem, Darmastadt, Germany). Drugs were added to cells every 4 days. The number of LCSs was assessed by counts under the microscope. LCSs diameters were determined using ImageJ software (NIH, Bethesda, MD).

Experiments were repeated at least three times and data are shown as mean ± SD of three independent experiments.

### Quantitative reverse transcription real-time PCR (Q-RT-PCR)

Total RNA was prepared as described [25]. Q-RT-PCR was performed using Power SYBR Green PCR Master Mix in an ABI Prism 7300 thermocycler (Applied Biosystems, Foster City, CA). Primers used in Q-RT-PCR are available upon request. Gene expression was normalised to GAPDH mRNA content. The relative amounts of mRNA were calculated by the comparative cycle threshold (CT) method [26]. At least three independent experiments were performed in triplicate and data are shown as mean ± SD.

### ELISA

Levels of secreted IL-6 were assessed by ELISA in culture supernatants (Human IL-6 Quantikine ELISA Kit, R&D Systems, Minneapolis, MN), according to manufacturer's instructions. The amount of IL-6 concentration was normalized for cell number after 48 h from plating. Three independent experiments were performed in triplicate and data are shown as mean ± SD.

### Antibodies and western blot

Antibodies used for Western Blot were from: i) Cell Signaling Technology (Denver, MA), anti-phospho-Akt (S473) (#4058), anti-Akt1 (#2938), anti-Akt2 (#2964), anti-phospho-GSK3-αβ (Ser21/9) (#9331), anti-GSK3 (#9338), anti-STAT3 (#9139), anti-phospho-STAT3 (Y705) (#9145), anti-phospho-IkKα/β (Ser176/180) (#2697), anti-IkB (#9242), anti-phospho-IkB (Ser32) (#2859), anti-PTEN (#9552), anti-p110α (#4255); ii) Santa Cruz Biotechnology (Santa Cruz, CA), anti-IkKα (sc-7218); iii) Sigma-Aldrich, anti-β-actin (clone AC-74, #A2228).

Antibodies used for immunostaining were selected according to previously published work: anti-CK7 (#35057) (Menarini, Florence, Italy), anti-CK34 (Enzo Diagnostics, Inc, Farmigale, NY), anti-pAkt Ser473 (#3787) (Cell Signaling Technology), anti-pSTAT3 Tyr705 (#9145) (Cell Signaling Technology), anti-IL-6 (AF-206-NA) (R&D Systems, Minneapolis, MN).

Protein preparation and Western blot analysis were carried out by standard methods [27]. Densitometric analysis of gel bands was carried out with ImageJ software (NIH).

### Luciferase assays

Cells were transfected with 1 μg of the NF-kB promoter/luciferase reporter plasmids plus 100 ng pRL Renilla as control vector (Promega, Madison, WI) with Fugene transfection reagent (Roche Molecular Biochemicals, Mannheim, Germany). Luciferase activity was rmeasured by Dual-Luciferase Reporter Assay System (Promega) using Thermo Scientific Varioskan Flash (Thermo, Waltham, MA). Basal luciferase activity was examined in cells transfected with an empty vector (pGL3 Basic Vector). Three independent transfections were performed for each construct in triplicate and data are shown as mean ± SD.

### RNA profiling analysis

RNA concentration was determined with Nanodrop spectrophotometer (Nano-Drop, Wilmington, Germany) and its quality was assessed with Agilent 2100 Bioanalyzer (Agilent Technologies, Santa Clara, CA). For each sample, 500 ng of total RNA were used to synthesize biotinylated cRNA with Illumina RNA Amplification Kit (Ambion, Austin, TX). Synthesis was carried out according to the manufacturers' instructions. cRNA concentration and the quality were assessed out as described above. From each sample, technical replicates were produced and 750 ng cRNA were hybridized for 18 h to Human HT-12_V3_0_R1 Expression BeadChips (Illumina, San Diego, CA) as described earlier [28]. Hybridized chips were washed and stained with streptavidin-conjugated Cy3 (GE Healthcare, Milan, Italy). BeadChips were dried and scanned with an Illumina Bead Array Reader (Illumina).

### Data analysis

Expression gene lists were analysed by Gene-Spring10.1 (Agilent Technologies) and DEGs were selected on the basis of fold-change and statistical significance. Lists were filtered using fold-change ≥1.5 and *p*-value ≤ 0.01 as thresholds in *T*-test. DEGs were used to evaluate the functional behaviour in terms of Biological Processes and Molecular Function, Development Function, Disease and Disorder. The degree of enrichment was evaluated to determine whether an observed level of annotation for a group of genes was statistically significant. In particular, for each term, a *q*-value was computed by the Hypergeometric test (*p* ≤ 0.05) and corrected using False Discovery Rate (FDR) [29]. The terms with a *q*-value exceeding the significance threshold were then selected as representative. Pathway analysis was performed using Ingenuity Pathway Analysis (IPA, Ingenuity Systems).

### Tumorigenicity assays

Tumorigenicity assay were performed as follows: NCI-H460 (1 × 10^6^), PEd/10 (3 × 10^6^), BEAS-2B (5 × 10^6^) cells and the corresponding derivatives were subcutaneously injected into the right flank of 6-week-old athymic nude mice (Charles River, Erkrath, Germany). Control BEAS-2B and derivatives grown as LCSs (4 × 10^3^ or 4 × 10^4^) were injected subcutaneously into NOD/Scid mice (Charles River). Tumor growth was monitored every 7 days and measured with an external caliper. Tumor volume was calculated as follows: tumor volume (mm^3^) = ½ (length × width^2^) [30].

### Chromatin immunoprecipitation

Chromatin immunoprecipitation (ChIP) was carried out with Imunoprecipitation Assay Kit (Upstate Biotechnology) according to the manufacturer's instruction. Approximately 10^7^ cells were fixed in 1% formaldehyde and 125 mM glycine. Cells were lysed and chromatin sonication was performed with a Bioruptor Diagenode model UCD300 to obtain an average fragment length of ~400 bp. Samples were precleared with protein A-G Sepharose and incubated overnight at 4°C with 2 μg of antibody anti-IgG or anti-STAT3, sc-482 (Santa Cruz Biotechnology). Input and immunoprecipitated DNAs were analyzed by PCR performed with AmpliTaq Gold DNA polymerase (Perkin-Elmer, MA, USA), resolved on 2% agarose gel and scanned using GelDoc (BioRad, Hercules, CA). Primers used were 5′-GCCGCTGTTTACAAGGACAC-3′ (forward) and 5′-CTAGTCAGCCACGGAAGTGC-3′ (reverse).

### Patients

Patient accrual was conducted according to internal Review Board of the INT Fondazione Pascale (Naples, Italy) (CEI 556/10 of 12/3/2010) and the study was approved by the internal Review Board of the AOU Mater Domini/University Magna Graecia (Catanzaro, Italy) in the meeting of 16/3/2011. All animal work was conducted according to the relevant Italian guidelines and was approved by the Internal Committee for Animal Study (CESA) of the Institute for Genetic Research on April 7th 2008 (CESA 10-08). Archive material from 104 patients diagnosed of NSCLC was collected from INT Fondazione Pascale (Naples, Italy) and University of Catanzaro. Median age was 64 year old (range 28–82). Among patients with clinical data available, women were 32 and males 72. Stage was known for 92 patients: 79 patients had stage I–II disease and 13 had stage III–IV disease. Grade was known for 75 patients: 52 cases were G1–G2 and 23 were G3–G4.

### TMA generation and immunohistochemistry

TMAs were constructed using Galileo TMA CK3500 Tissue Microarrayer (ISE TMA Software, Integrated System Engineering). Immunostaining was performed using the avidin-biotin-peroxidase method (Novocastra, Newcastle, UK) on formalin-fixed, paraffin-embedded tissues as described [19, 27, 31]. Positive immunohistochemical scores were selected on the basis of established criteria: pAkt and IL-6 was scored positive when >10% of tumor cells were positive, respectively [32, 33]; pSTAT3 was scored positive when >5% of tumor cells were positive [34].

For each one immunohistochemical round a negative control has been included by replacing the primary antibody with solvent. All controls gave satisfactory results. Stained TMA sections were evaluated by an expert pathologist (R.F.).

### Statistical analysis

Data presented are the means ± SD of at least 3 independent assays (number and diameter of spheres, ELISA assay, Luciferase assay, Q-RT-PCR). Number and diameter of spheres, fold increase were analyzed by Student's *t*-test and ANOVA using the statistical software GraphPad Prism 4. Fischer's exact test was used to assess the correlation between pAkt, pSTAT3 and IL-6 expression. Data analysis and summarization were conducted using SPSS 20 (SPSS Inc., Chicago, IL, USA). Significance was set with *p* value of at least at *p* ≤ 0.05. In the figures the number of asterisks corresponds to *p* ≤ 0.05 (*), *p* ≤ 0.01 (**), *p* ≤ 0.001 (***) and *p* ≤ 0.0001 (****), respectively.

## SUPPLEMENTARY FIGURES AND TABLES








