# Supplementary material for: The Akt1/IL-6/STAT3 pathway regulates growth of lung tumor initiating cells
Source: Oncotarget. 2015 Oct 13;6(40):42667–86. doi: 10.18632/oncotarget.5626 (PMC4767462; doi:10.18632/oncotarget.5626)
Supplement: Supplementary file 1 [file oncotarget-06-42667-s001.pdf]

SUPPLEMENTARY FIGURES AND TABLES

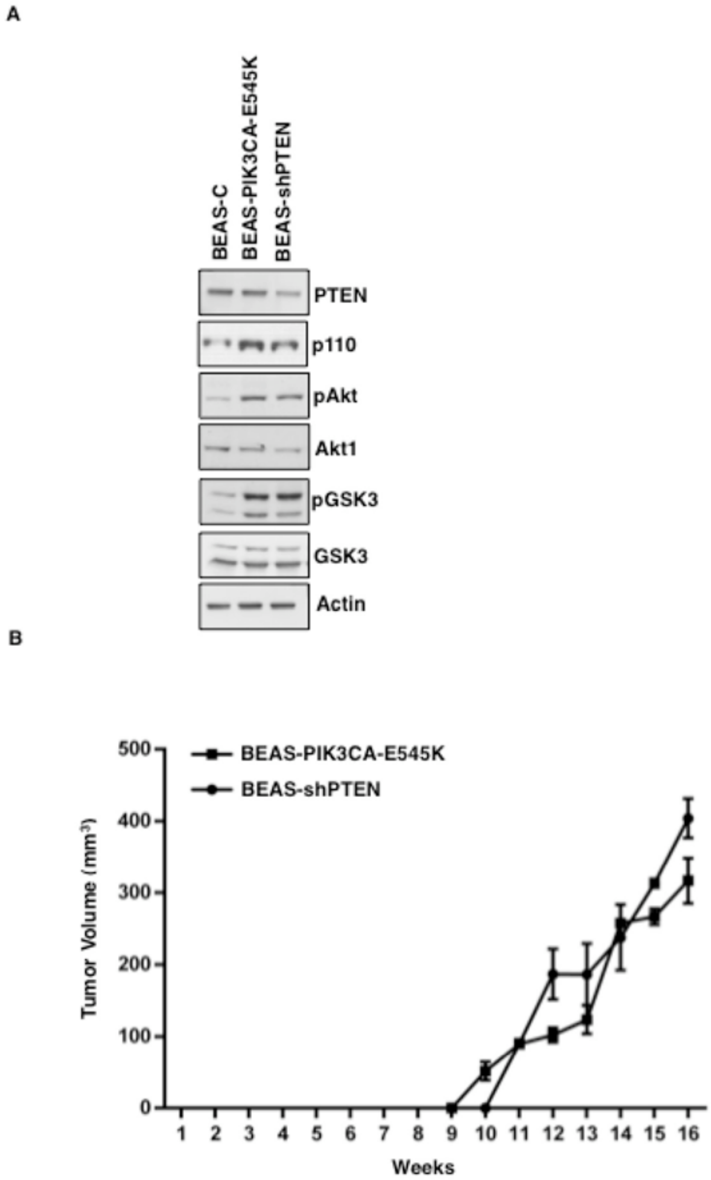

Supplementary Figure S1: Activation of PTEN/PI3K/Akt1 pathway promotes tumorigenesis in BEAS-2B cells. A. Immunoblot analysis of PTEN, p110, phosphorylated and total Akt1, phosphorylated and total GSK3 and actin in BEAS-C, BEAS-PIK3CA and BEAS-shPTEN cells. **B.** Tumor growth promoted by BEAS-PIK3CA and BEAS-shPTEN cells (5×10^6) grown in adherent condition injected in NOD/SCID mice ($n = 8$/group).

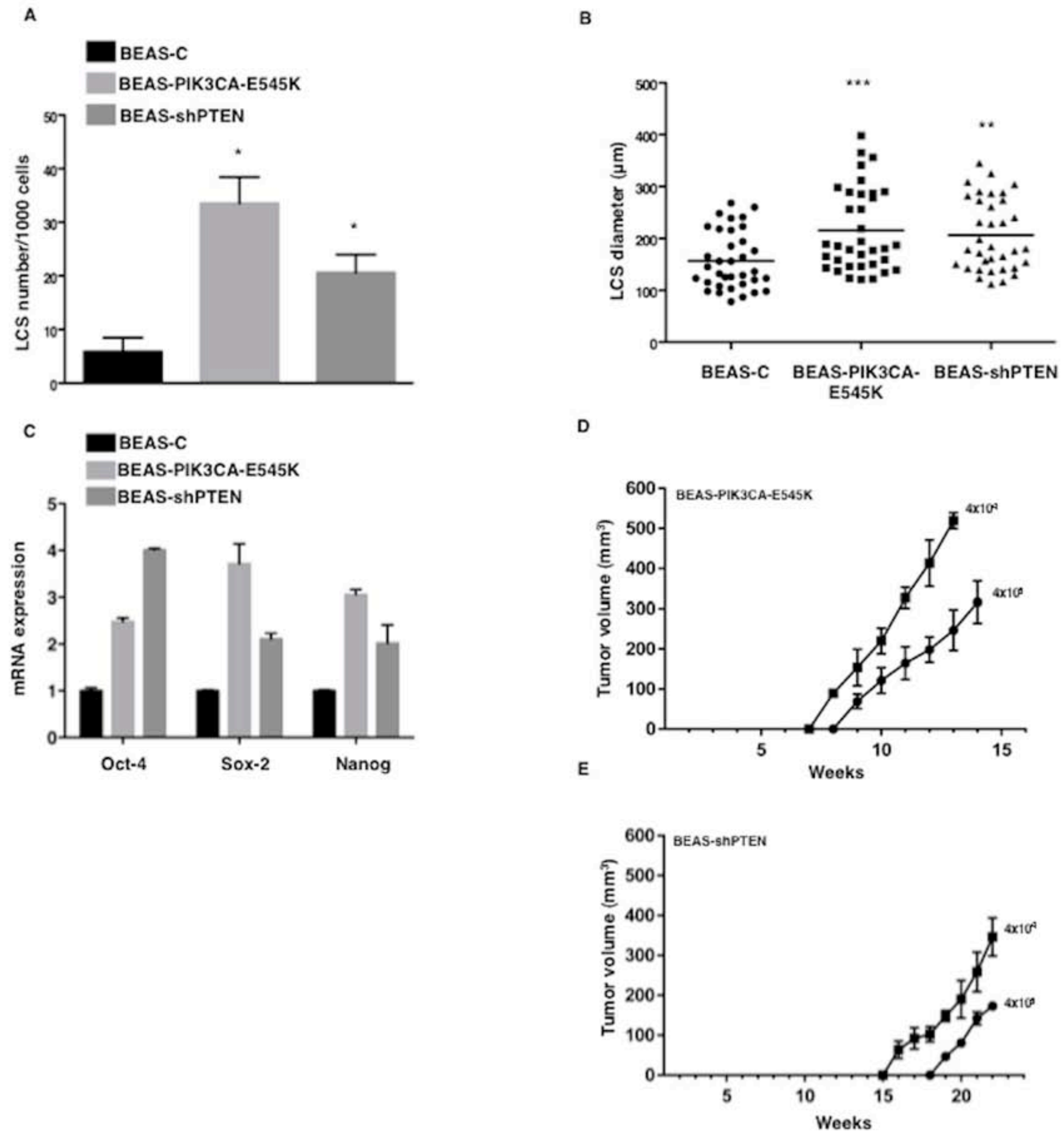

Supplementary Figure S2: Activation of PTEN/PIK3CA/Akt1 pathway regulates formation and maintenance of LCSs. A. Number of LCSs generated from BEAS-C, BEAS-PIK3CA and BEAS-shPTEN cells. $^{*}p < 0.05$. **B.** Phase-contrast microscopy analysis of size distribution (μm) of LCSs generated from BEAS-C, BEAS-PIK3CA and BEAS-shPTEN cells $^{***}p < 0.001$, $^{**}p < 0.01$. **C.** Relative mRNA expression of stemness genes by Q-RTPCR in BEAS-C cells and derivatives (mean ± SD). **D.** Tumor growth promoted by single cell suspensions of LCSs generated from BEAS-PIK3CA (4×10^3, 4×10^4 cells) injected into the flank of NOD/SCID mice ($n = 8$/group). **E.** Tumor growth promoted by single cell suspensions of LCSs generated from BEAS-shPTEN (4×10^3, 4×10^4 cells) injected into the flank of NOD/SCID mice ($n = 8$/group).

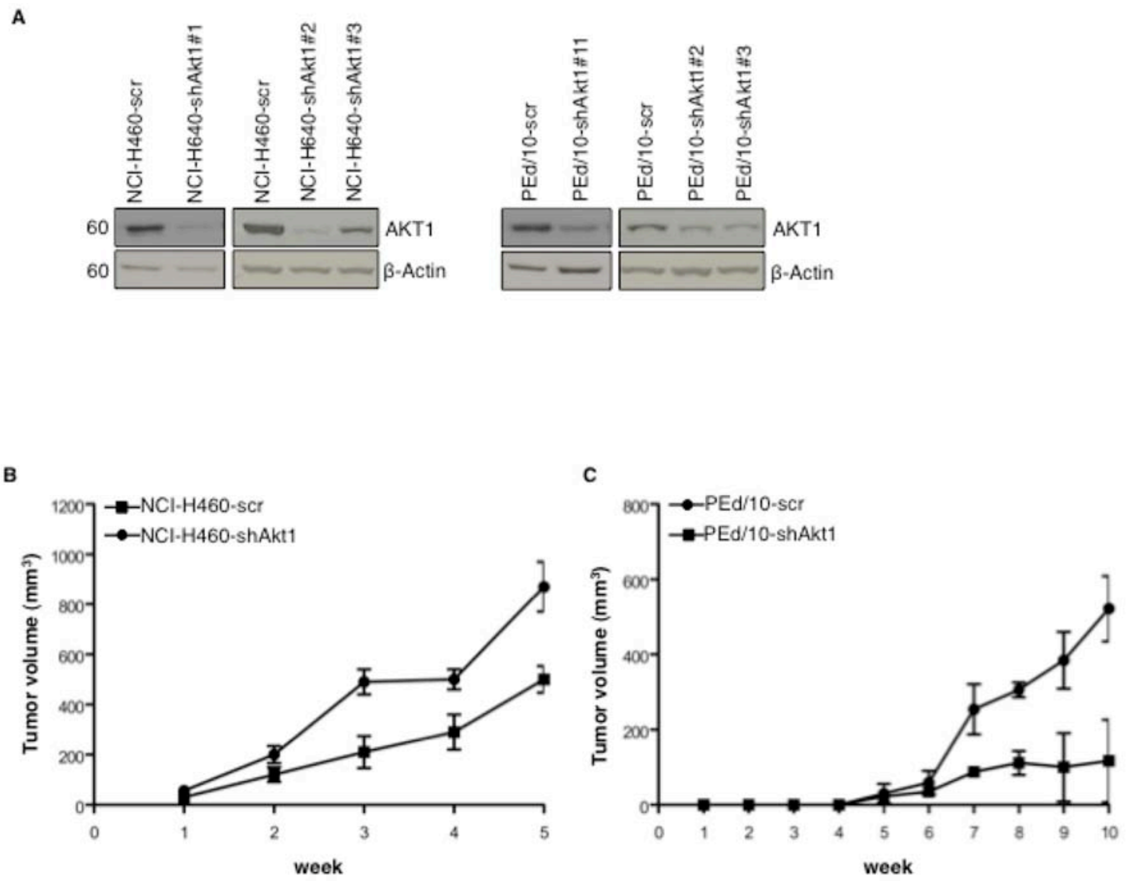

Supplementary Figure S3: Akt1 suppression reduces *in vivo* growth of established and primary NSCLC cells. A. Left, immunoblot of Akt1 in NCI-H460-scr cells and the corresponding NCI-H460-shAkt1 cells (clones #1, #2, #3); right, immunoblot of Akt1 in PEd/10-scr and PEd/10-shAkt1 cells (clones #1, #2, #3). **B.** Tumor growth promoted by NCI-H460-scr and NCI-H460-shAkt1 cells grown in adherent conditions injected into nude mice (*n* = 5/group). **C.** Tumor growth promoted by PEd/10-scr and PEd/10-shAkt1 cells grown in adherent conditions injected into nude mice, (*n* = 5/group).

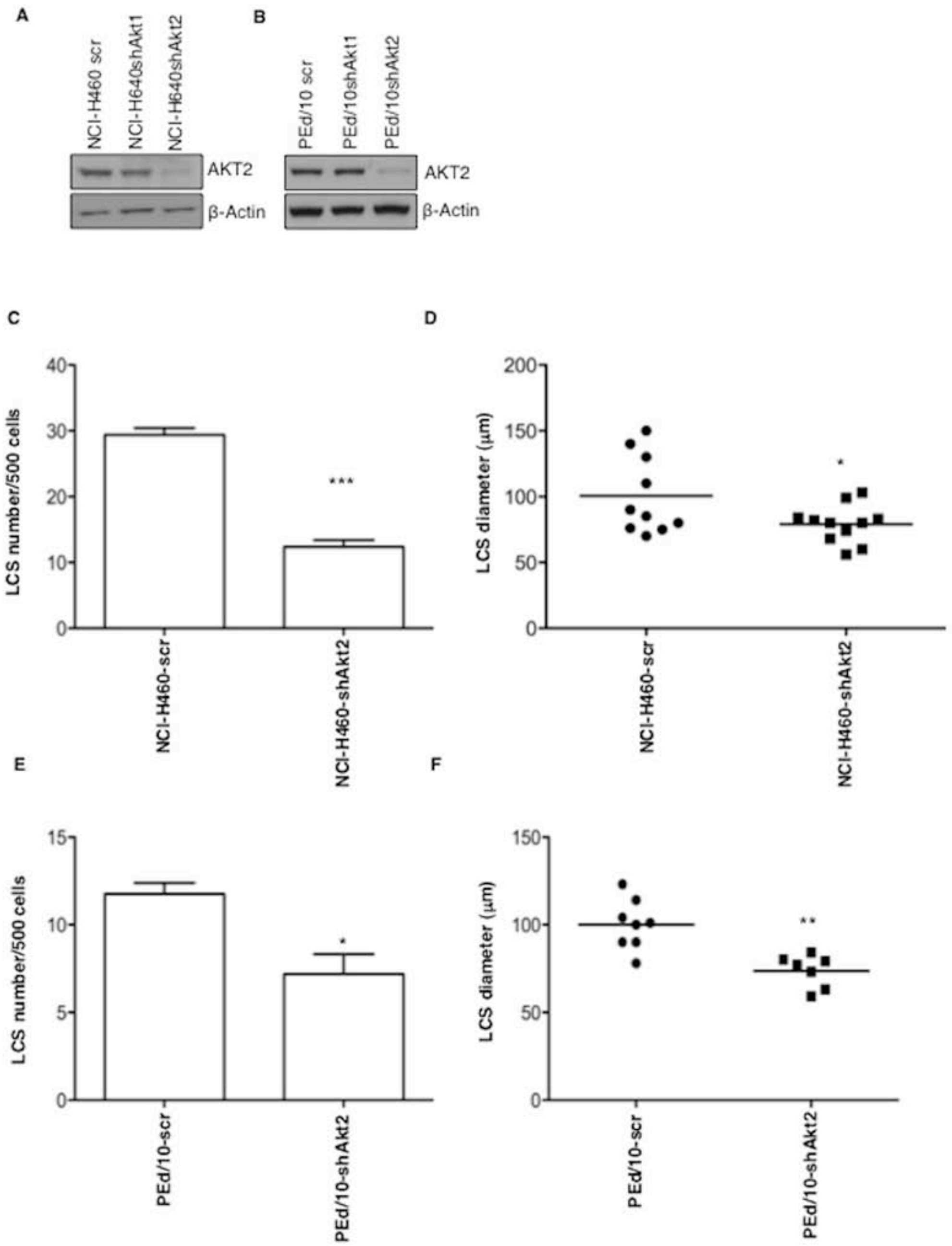

Supplementary Figure S4: Relative effects of Akt1 and Akt2 on the formation and maintenance of NSCLC LCSs. **A.** Immunoblot of Akt2 in NCI-H460-scr and the corresponding shAkt1- and shAkt2-interfered cells. **B.** Immunoblot of Akt2 in PEd/10-scr cells and the corresponding shAkt1- and shAkt2-interfered cells. **C.** Number of primary LCSs generated from NCI-H460-scr or from the corresponding cells interferred for Akt2. $^{**}p < 0.01$. **D.** Phase-contrast microscopy analysis of size distribution (μm) of LCSs generated from NCI-H460-scr cells or from the corresponding cells interferred for Akt2 $^{*}p < 0.05$. **E.** Number of primary LCSs generated from PEd/10-scr cells or from the corresponding cells interferred for Akt2. $^{**}p < 0.01$. **F.** Phase-contrast microscopy analysis of size distribution (μm) of LCSs generated from PEd/10-scr cells or from the corresponding cells interferred for Akt2. $^{**}p < 0.01$. Data are presented from replicate analysis as the mean ± SD.

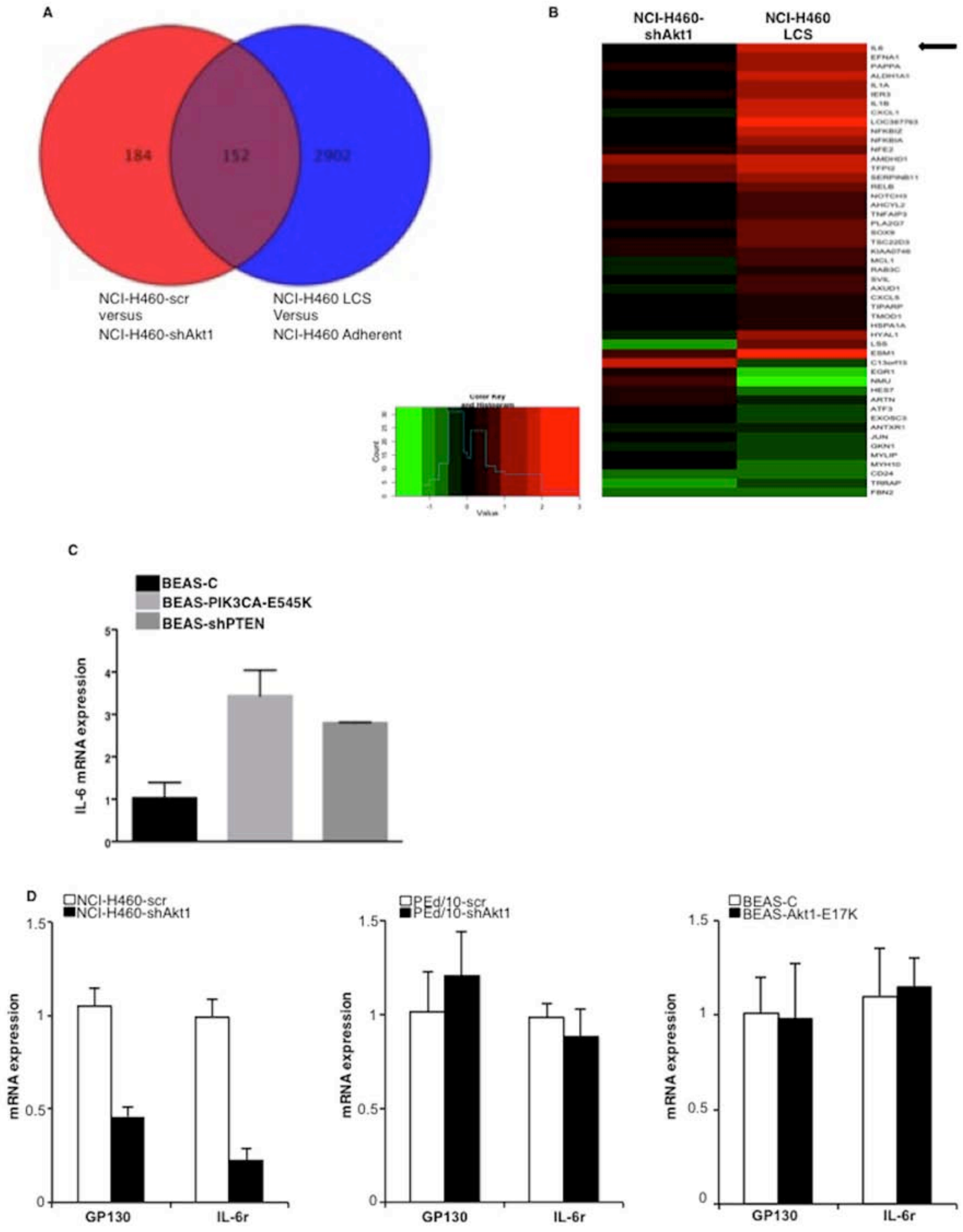

Supplementary Figure S5: Identification of Akt1-regulated genes that are differentially expressed in NSCLC LCSs. A. Venn diagram showing the comparison between genes that are differentially expressed in Akt1-interferred NCI-H460 cells and genes that are differentially expressed in LCSs from NCI-H460 cells. **B.** Heat-map representation of the comparison between genes that are differentially expressed in Akt1-interferred NCI-H460 cells and in LCSs generated from NCI-H460 cells. **C.** Q-RT-PCR analysis of mRNA expression of IL-6 in BEAS-C and derivative cells. **D.** Q-RT-PCR analysis of mRNA expression of GP130 and IL-6r in NCI-H460 (left), PEd/10 (middle), BEAS-C (right) and derivative cells.

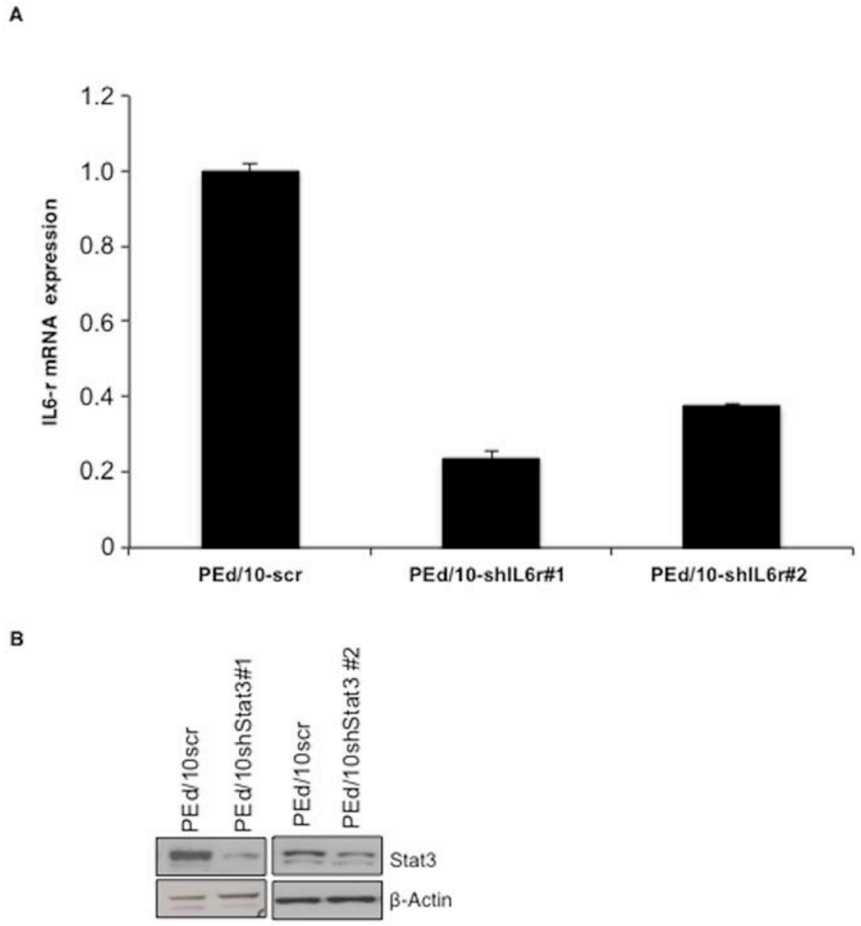

Supplementary Figure S6: Generation of primary NSCLC cells interfered for IL-6r and STAT3. A. Q-RT-PCR analysis of IL-6r expression in PEd/10 cells and in two different PEd/10-shIL-6r cells (clones #1, #2). **B.** Immunoblot analysis of STAT3 in PEd/10-scr and PEd/10-shSTAT3 cells (clones #1, #2).

Supplementary Table S1:

See Supplementary File S1

Supplementary Table S2:

See Supplementary File S2

Supplementary Table S3:

See Supplementary File S3

Supplementary Table S4: Analysis of phosphorylated Akt, phosphorylated STAT3 and IL-6 expression in NSCLC

	pAkt negative[a]	pAkt positive[a]	Total
**IL-6 negative [b]**	43	16	59
**IL-6 positive [b]**	19	16	35
**Total**	**62**	**32**	
**pSTAT3 negative [c]**	43	18	61
**pSTAT3 positive [c]**	19	14	33
**Total**	**62**	**32**	

[a]Akt activation was evaluated with phospho-specific antibodies (pS473) and scored as negative (<10% of the tumour cells with weak, focal immunopositivity or absence of staining) and positive (>10% of tumour cells with strong or diffuse immunopositivity).
[b]IL-6 was evaluated with specific antibodies and scored as negative (<10% of the tumour cells with weak, focal mmunopositivity or absence of staining) and positive (>10% of tumour cells with strong or diffuse immunopositivity).
[c]STAT3 activation was evaluated with phospho-specific antibodies (Y705) and scored as negative (<5% of the tumour cells with weak, focal immunopositivity or absence of staining) and positive (>5% of tumour cells with strong or diffuse immunopositivity). $n = 94$, $p = 0.044$
